# Supplementary material for: Identification and Validation of a Previously Missed Mutational Signature in Colorectal Cancer
Source: bioRxiv. 2025 Jun 7:2025.06.06.658321. Preprint. [Version 1] doi: 10.1101/2025.06.06.658321 (PMC12258970; doi:10.1101/2025.06.06.658321)

Supplementary Figure 1

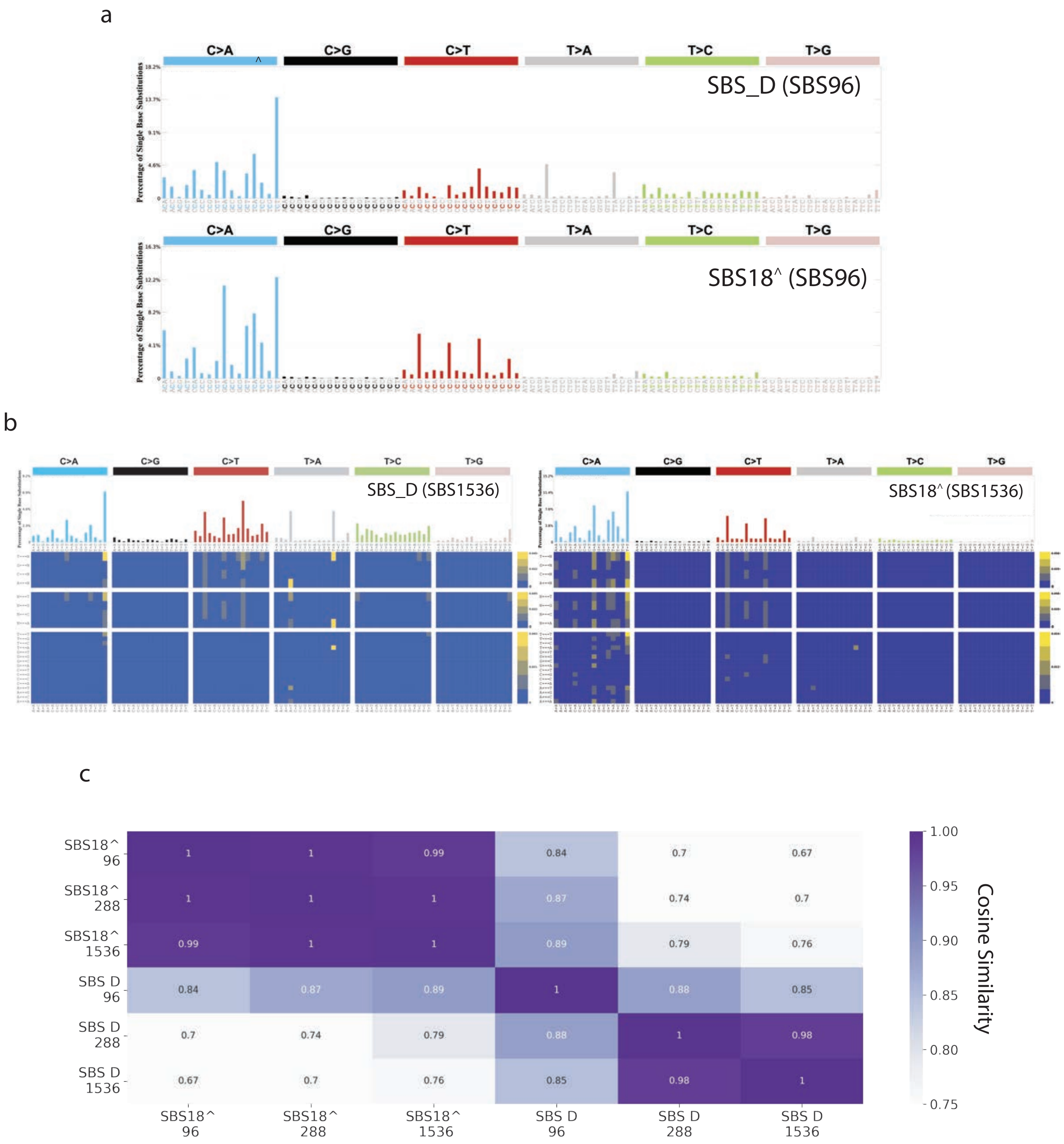

Supplementary Figure 2

a

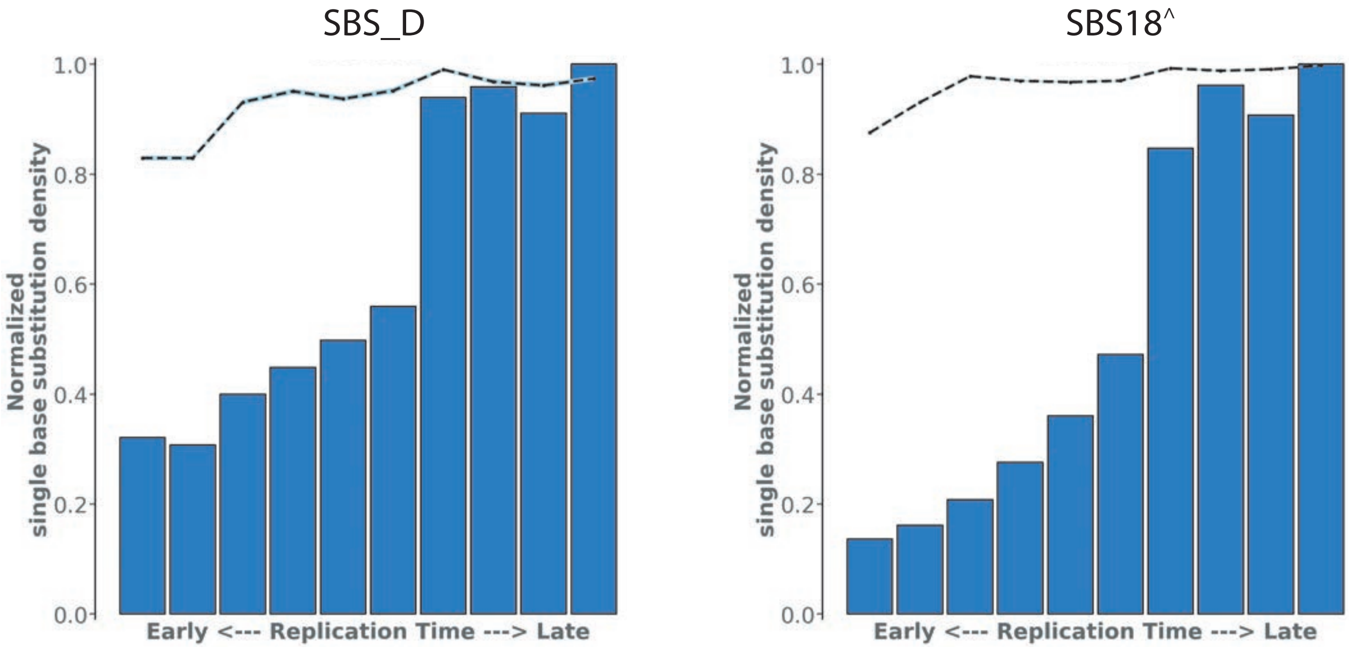

b

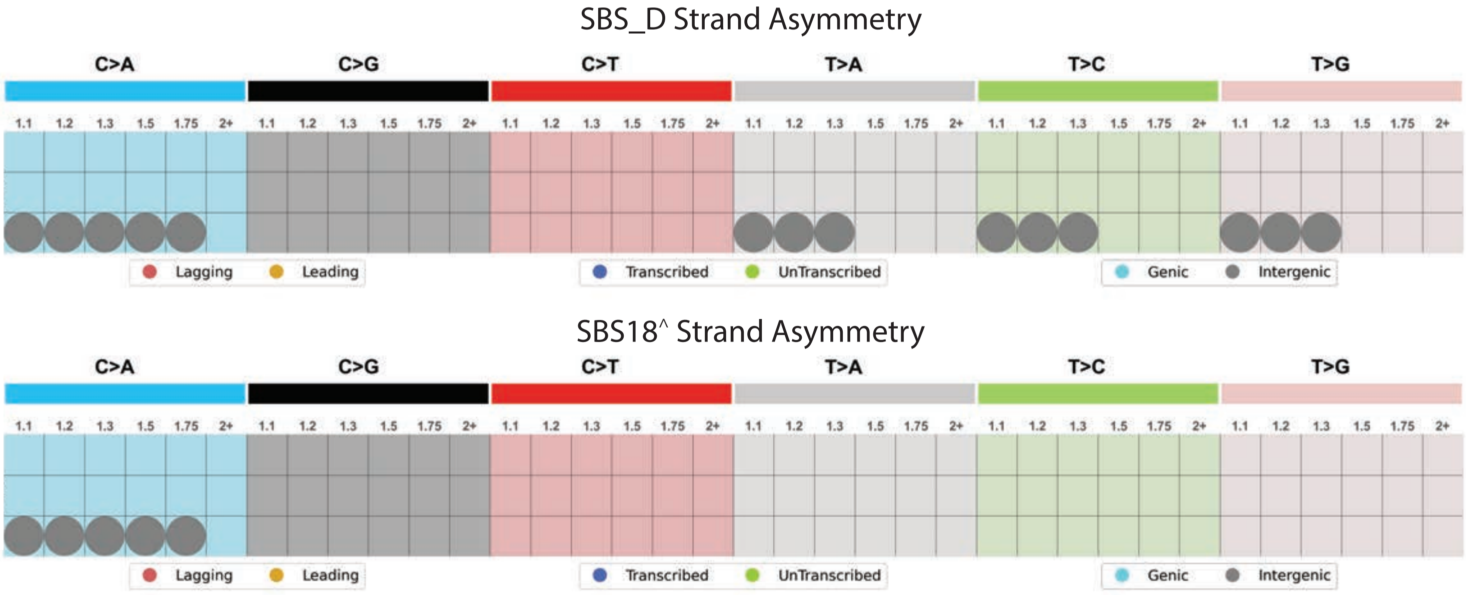

Supplementary Figure 3

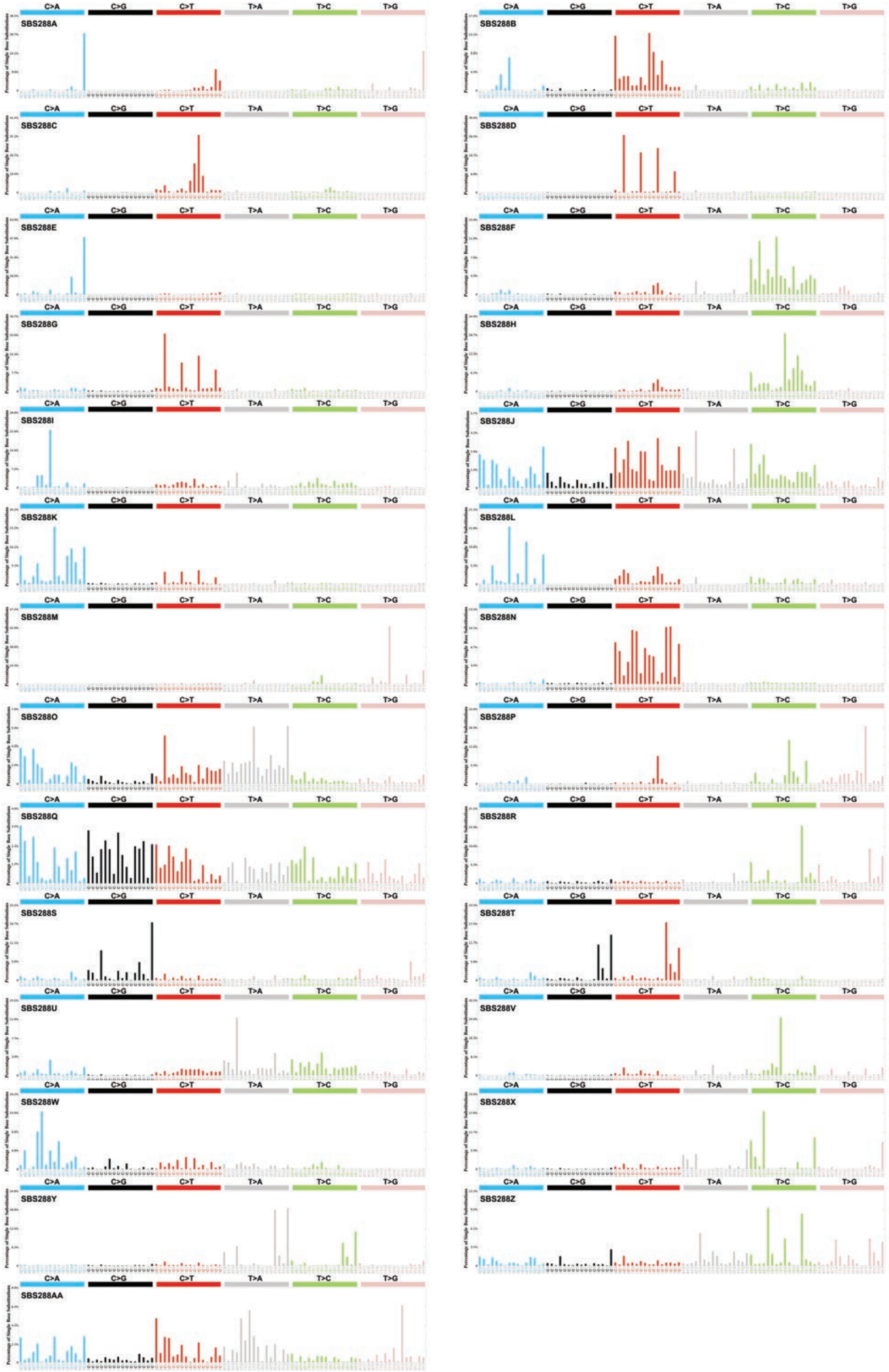

Supplementary Figure 4

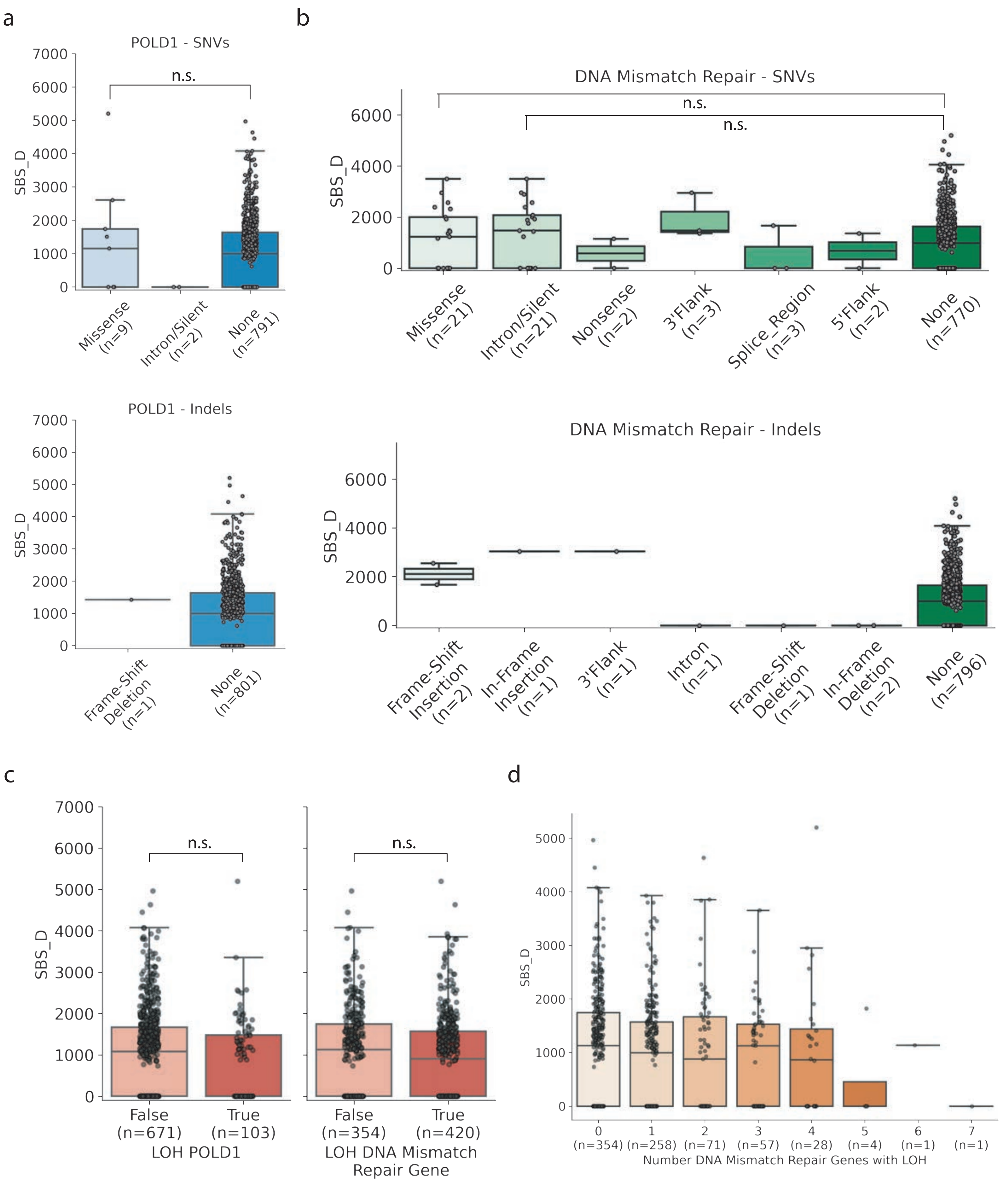

Supplementary Figure 5

a

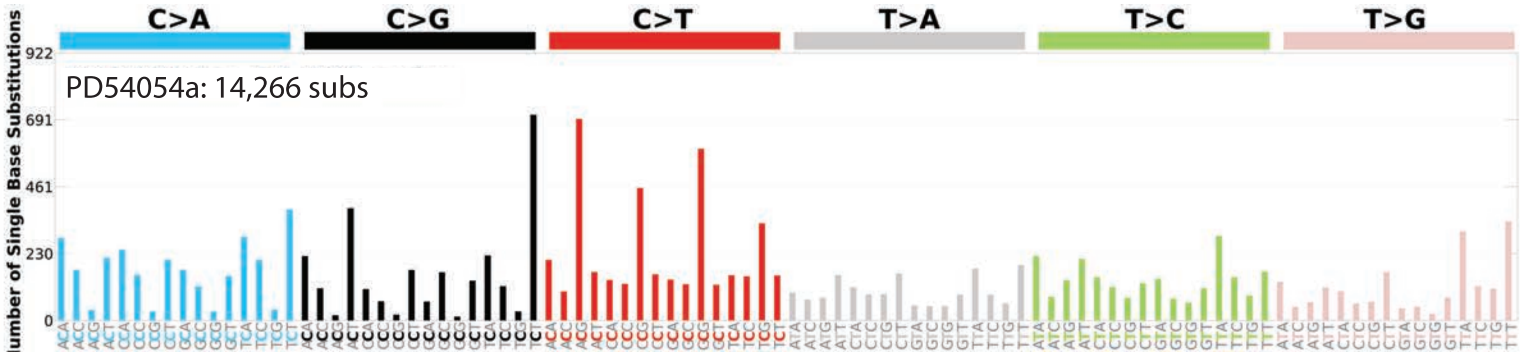

b

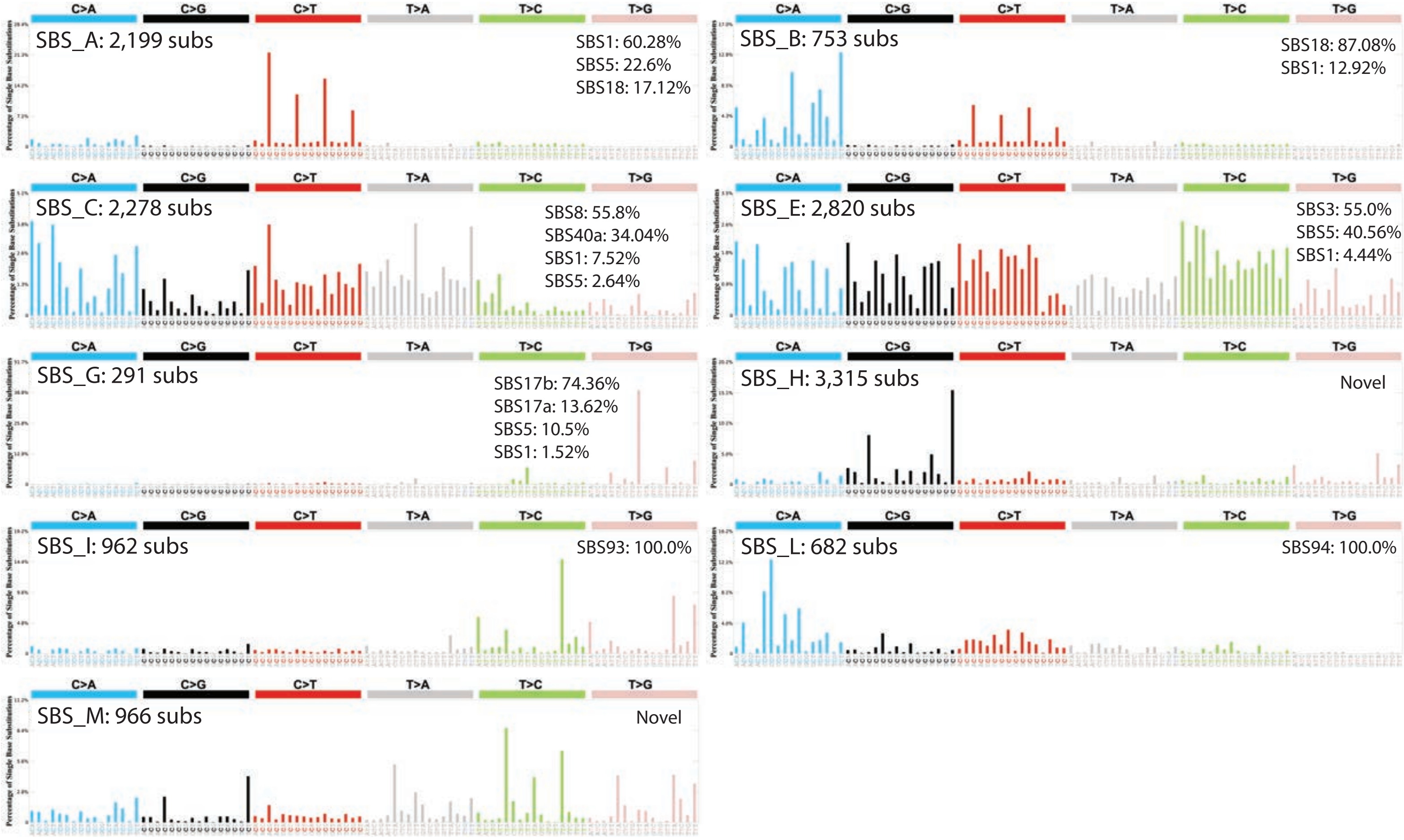

Supplementary Figure 6

SBS_D status

Adjusted by age, sex, tumor subsite, and purity (with the exception of GEL)

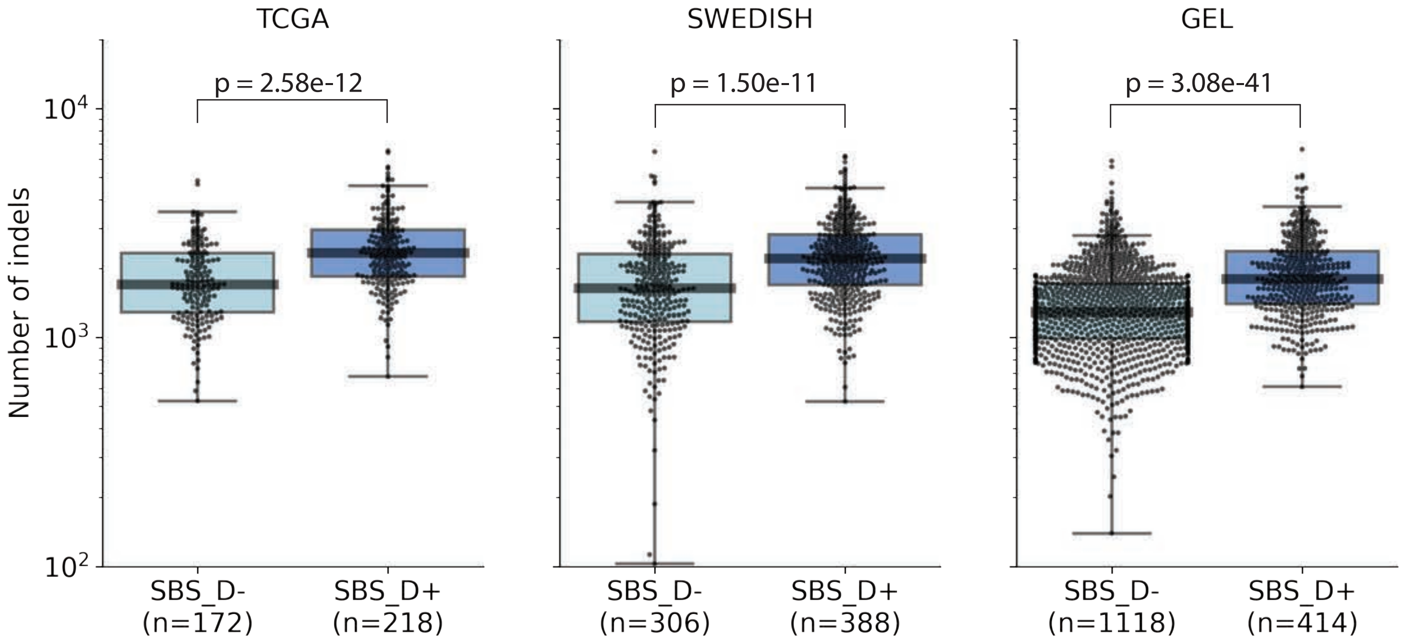

Supplement: Supplement 1 — Supplementary Figure 1: SBS18^ and SBS_D are recapitulated across signature extractions from multiple contexts in the Mutographs colorectal cancer cohort. (a) SBS_D-like (top) and SBS18^-like (bottom) signatures extracted from the Mutographs colorectal cancer cohort using the SBS-96 context. (b) Same as (a), but signatures were extracted using the SBS-1536 context. (c) Cosine similarity between the SBS18^-like signatures and SBS_D-like signatures extracted from each of the SBS-96, SBS-288, and SBS-1536 contexts. All signatures were collapsed to the SBS-96 context for comparison. Supplementary Figure 2: SBS_D and SBS18^ exhibit similar patterns for some topographical features. (a) Replication timing patterns for SBS_D (left) and SBS18^ (right). Blue bars represent deciles (10% bins) of the replication timing signal; the dashed line indicates the distribution of simulated somatic mutations. (b) Strand asymmetry profiles for SBS_D (top) and SBS18^ (bottom). Circles indicate statistically significant biases (Fisher’s exact test with Benjamini-Hochberg multiple testing correction), with circle color representing the genomic context. Both signatures show enrichment in intergenic regions (gray circles), while no significant differences were observed between leading versus lagging or between transcribed versus un-transcribed strands. Numbers indicate odds ratios for each comparison. Supplementary Figure 3: SBS_D is not extracted when DNA repair proficient and deficient samples are combined. De novo mutational signatures extracted from all the Mutographs colorectal cancer samples (n=981), without stratifying by DNA repair status. Extraction was performed using the SBS-288 context; for simplicity, signatures are shown here in the SBS-96 context. Supplementary Figure 4 SBS_D activity is not associated with mutations in POLD1 or mismatch repair genes. (a) SBS_D activity in samples with single nucleotide variants (SNVs, top) and indel mutations (bottom) in POLD1. Statistical sig [file media-1.pdf]
